# Supplementary material for: A high-resolution mRNA expression time course of embryonic development in zebrafish
Source: eLife. 2017 Nov 16;6:e30860. doi: 10.7554/eLife.30860 (PMC5690287; doi:10.7554/eLife.30860)
Supplement: Supplementary file 6. [file elife-30860-supp6.zip › biolayout-clusters-files/Cluster217-genes.html]

Cluster217


# Cluster217: Genes

| Ensembl ID | Gene Name | Chr | Start | End | Biotype |
| --- | --- | --- | --- | --- | --- |
| ENSDARG00000015978 | COX5B (1 of many).1 | 10 | 42546390 | 42550144 | protein\_coding |
| ENSDARG00000104537 | cox7c | 5 | 47105169 | 47106791 | protein\_coding |
| ENSDARG00000039346 | ndufa5 | 4 | 4921836 | 4924049 | protein\_coding |
| ENSDARG00000056583 | ndufs6 | 19 | 28696978 | 28699654 | protein\_coding |
| ENSDARG00000021346 | pdhb | 22 | 10172144 | 10182820 | protein\_coding |
| ENSDARG00000055976 | pecr | 11 | 30108258 | 30117663 | protein\_coding |
| ENSDARG00000069360 | ppp3r1b | 1 | 50913990 | 50947637 | protein\_coding |
